# E-cadherin expression promotes tumor growth via KLRG1-dependent pathways

**DOI:** 10.1093/jimmun/vkag046

**Published:** 2026-04-24

**Authors:** Jacob A Myers, Alexander R D Jordon, Samantha M Borys, KhanhLinh Dinh, Laurent Brossay

**Affiliations:** Division of Biology and Medicine, Department of Molecular Microbiology and Immunology, Brown University, Providence, RI, United States; Division of Biology and Medicine, Department of Molecular Microbiology and Immunology, Brown University, Providence, RI, United States; Division of Biology and Medicine, Department of Molecular Microbiology and Immunology, Brown University, Providence, RI, United States; Division of Biology and Medicine, Department of Molecular Microbiology and Immunology, Brown University, Providence, RI, United States; Division of Biology and Medicine, Department of Molecular Microbiology and Immunology, Brown University, Providence, RI, United States

**Keywords:** CD8^+^ T cells, E-cadherin, tumor, KLRG1, NK cells

## Abstract

Transformed cells frequently adapt to immune pressure by modulating expression of ligands for PD-1 and CTLA-4. Despite the success of therapies targeting these interactions, only a minority of patients experience a clinical response, highlighting the critical need for alternative targets. Prior work from our group showed that the inhibitory receptor KLRG1 binds to tumor-derived E-cadherin. However, the therapeutic potential of targeting KLRG1 signaling remains incompletely understood. To address this, we generated a library of cancer cell lines engineered to express varying levels of E-cadherin. Across multiple models, we demonstrate that E-cadherin expression renders tumors more aggressive in vivo. Using an RMA-S cell line possessing a mutated E-cadherin that abrogates KLRG1 binding while preserving cadherin homotypic interactions, we demonstrate that the protumor effect of E-cadherin expression is largely mediated by KLRG1. To further dissect the cellular targets of KLRG1 signaling, we generated KLRG1^fl/fl^ mice and performed lineage-specific deletion. Surprisingly, we discovered that KLRG1 expression on CD8^+^ T cells, rather than on natural killer cells, impairs the immune control of B16-F10 E-cadherin^+^ tumors. Taken together, these results reveal an underappreciated protumor role for E-cadherin and highlight KLRG1 as a promising target for future checkpoint blockade strategies, particularly in tumors retaining epithelial features.

## Introduction

It is widely recognized that features on transformed cells can dictate both cell-intrinsic and extrinsic changes that lead to disease progression. One common alteration associated with the growth of metastatic cancer is the dysregulation of cadherin expression. Cadherins constitute a large family of evolutionarily conserved glycoproteins characterized by an extracellular domain that mediates cell-cell interactions, a transmembrane domain, and a cytoplasmic domain linked to the cytoskeleton. These proteins facilitate calcium-dependent homophilic binding between cells and exhibit diverse expression patterns associated with morphogenetic processes. Among them, classical cadherins, including E-cadherin, N-cadherin, and VE-cadherin, are Ca^2+^-dependent adhesion molecules expressed in nearly all tissues. E-cadherin is predominantly expressed in epithelial cells, localizing to adherens junctions, while N-cadherin is also present in cardiac adherens junctions. Notably, N-cadherin knockout mice experience early embryonic lethality due to severe cardiac defects, and E-cadherin knockout mice exhibit even earlier embryonic lethality.^1^^,^^2^ However, during tumorigenesis, cells lose E-cadherin expression, which promotes the growth of malignant tissue in a process termed the epithelial-to-mesenchymal transition (EMT).^3–6^ Though the EMT is a well-established driver of carcinoma progression, loss of E-cadherin is not a prerequisite for metastatic tumor growth. During the mesenchymal-to-epithelial transition (MET), in which transformed cells establish secondary outgrowths after leaving the primary tumor, E-cadherin is commonly re-expressed, and this phenomenon has been reported to correlate with poor overall patient survival.^7–12^ Moreover, preclinical studies have revealed a causal role for E-cadherin in accelerating cancer progression in multiple tumor models.^13^^,^^14^ Nevertheless, the tumor-promoting effects of E-cadherin remain incompletely characterized, and the influence of its re-expression on antitumor immunity is not fully understood.

Previously, our laboratory and others identified E-cadherin as a ligand for the inhibitory receptor KLRG1.^15^^,^^16^ KLRG1 is expressed by natural killer (NK) cells and differentiated T cells and raises the cellular activation threshold while diminishing immune responses in these populations.^17–21^ In naive mice, approximately 30% of NK cells and 2% of T cells express KLRG1; however, expression on both populations increases substantially following activation and differentiation.^17^^,^^19^^,^^22^ In humans, the fraction of KLRG1-expressing lymphocytes is substantially higher, with 50% to 80% of NK cells and 20% to 30% of T cells expressing this receptor, depending on age.^23^^,^^24^ Though the ability of KLRG1 to dampen lymphocyte activation following recognition of E-cadherin is relatively well established, the translational value of inhibiting KLRG1-E-cadherin interactions has been incompletely explored. Intriguingly, the expression of E-cadherin has been found to correlate with responsiveness to immune checkpoint inhibitor therapy, implicating a role for cadherins in therapeutic responses in humans.^25^ Previously, our group demonstrated a robust synergistic effect of immune checkpoint inhibitor therapies targeting KLRG1 and PD-1 using a model of E-cadherin^+^ melanoma in vivo.^26^ Nevertheless, the effects of KLRG1 disinhibition on antitumor immunity remain poorly understood, thus hindering the development of KLRG1-targeted immunotherapies and other novel treatments for cancer.

To investigate this topic, we developed a library of tumor cell lines engineered to express varying degrees of E-cadherin expression and differing KLRG1-binding affinities. Across all cell lines, we demonstrate that E-cadherin promotes tumor growth through KLRG1-dependent and KLRG1-independent mechanisms. Using immunodeficient animals, we uncover variable sensitivity to adaptive and innate immune control across our E-cadherin^+^ cell line library, highlighting a tumor-specific effect of E-cadherin expression in our models. Finally, using tissue-specific in vivo deletion, we demonstrate that KLRG1 deficiency in CD8^+^ T cells enhances immune control of E-cadherin^+^ B16-F10 melanoma. Together, these results reveal a strong KLRG1-dependent protumor effect of E-cadherin and demonstrate that abrogating KLRG1 signaling in CD8^+^ T cells can alleviate the tumor burden in our metastatic melanoma model.

## Methods

### Mice

C57BL/6 (RRID: IMSR_JAX:000664), C57BL/6-Rag1^−/−^ (RRID: IMSR_JAX:002216), and Balb/c (RRID: IMSR_ORNL: BALB-CRL) mice were purchased from the Jackson Laboratory. Balb/c-Rag1^−/−^ (RRID: IMSR_JAX:036333) mice were purchased from the Jackson Laboratory and maintained in-house. KLRG1^−/−^ (RRID: ISMR_JAX:038186) were generated in-house and have been described previously.^26^ C57BL/6-Tg(Cd8a-cre)1Itan/J (E8i-cre, but referred to as CD8-cre) mice (RRID: IMSR_JAX:00876) were crossed to KLRG1^fl/fl^ mice to obtain CD8-Cre KLRG1^fl/fl^ mice. Ncr1-Cre mice were a gift from Eric Vivier (Aix-Marseille University) and maintained in-house until bred with KLRG1^fl/fl^ mice to obtain Ncr1-Cre KLRG1^fl/fl^ animals. KLRG1^fl/fl^ animals were generated using CRISPR/Cas9 mediated homology-directed repair and loxP sites were inserted on each side of the targeted exon 3. Two single guide RNAs (sgRNAs) were generated to target *Klrg1* 5′ upstream of exon 3 in the surrounding intron: 46_Klrg1_sgRNAup1: ACATATGACCTCAAGCTGAA and 47_Klrg1_sgRNAup2: ATTGTGGACCATTCAGCTTG. In addition, 2 sgRNAs were generated to target 3′ downstream of exon 3 in the surrounding intron: 48_Klrg1_sgRNAdw1: TAGTTAAGATGTCACCTAAA and 49_Klrg1_sgRNAdw2: CTTAACTATGTAGTCCAGAC. sgRNAs were selected using the Benchling CRISPR Guide RNA design tool (RRID: SCR_013955). Cas9/plasmid injection was performed on C57BL/6NJ zygotes (RRID: IMSR_JAX: 005304). Nonhomologous end joining led to a 418-bp deletion of the sequence between sgRNAs, resulting in loss of exon 3 of *Klrg1*. Founders were genotyped via long-range polymerase chain reaction and sequenced at the *Klrg1* locus. Long-range polymerase chain reaction (LR-PCR) primers were as follows: 56_Klrg1_dwR1: CAGCCATCGATAATGAGATCTG; 59_Klrg1_upF2: TGACCTCATGAACTCTGTGAGC; 63_Klrg1_seqF: ACGAGGAATGGTAGCCACTG. 5′ LR-PCR was sequenced with #59 and #63. 3′ LR-PCR was sequenced with #59 and #56. LR-PCR products were cloned into a TA vector and sequenced with #56 and #59 to confirm that the two LoxP sites were in cis. The resulting mouse line was backcrossed to C57BL/6J (RRID: IMSR_JAX: 000664) mice several times. Genotyping primers for KLRG1^fl/fl^ animals generated were forward (CACTTGGTCAGAATCCATGCCT) and reverse (GGATGGGGCAGCTGGGGCAGTG). All tumor counts were performed blind. Six- to 12-wk-old sex- and age-matched mice were used in this study, which was conducted in an AAALAC-accredited, pathogen-free facility.

### Sex as a biological variable

This study examined male and female animals, and no significant differences were observed between sexes.

### Mouse infections

Mice were infected intraperitoneally with 5 × 10^4^ plaque-forming units of MCMV-RVG102. MCMV-RVG102 stocks were developed in vivo as previously described.^27^

### Cell lines and culture conditions

B16-F10 and B16-F10 E-cadherin (a gift from Five Prime Therapeutics) and RMA-S were previously described.^26^ 4T1 cells were purchased from ATCC (Cat# CRL-2539, RRID: CVCL_0125). The BWZ.36.KLRG1 reporter cell line was described previously.^16^ Plat-E (RRID: CVCL_B488) and B16-F10 cells were cultured in DMEM with L-glutamine (4 mM), Glucose (4500 mg/L), and sodium pyruvate (Thermo Fisher Scientific; Cat# SH30243.01). All other cell lines were cultured in RPMI medium with L-glutamine (2.05 mM), glucose (4500 mg/L), and sodium pyruvate (Thermo Fisher Scientific; Cat# SH30243.01). All media contained 8% fetal bovine serum (FBS) (Atlanta Biologics; Cat# S11550; or Gibco; Cat# 10437-028), penicillin, streptomycin, and glutamine (100 U/mL; Gibco; Cat# 10378-016), and 48 μM β-mercaptoethanol (Gibco; Cat# 21985-023), and cells were cultured in 5% CO_2_ at 37 °C. Cell lines were tested for mycoplasma contamination once per year and routinely tested negative. Cell lines were authenticated by flow cytometry.

### BWZ.36.KLRG1 reporter cell line assay

RMA-S or RMA-S E-Cad–derived cells (3 × 10^4^) were incubated with BWZ.36.KLRG1 reporter cells (5 × 10^4^) overnight. After incubation, cells were washed twice with phosphate-buffered saline (PBS)-1% FBS and fixed for 10 min at 4 °C using 10% buffered formalin phosphate (Fisher Scientific; Cat# SF100) and 25% glutaraldehyde (MilliporeSigma; Cat# 354400). Cells were washed twice with PBS and treated with X-Gal substrate (Invitrogen; Cat# 15520034) and incubated at 37 °C for 6 h. Cells were imaged with an Olympus DP70.

### Generation of E-cadherin knockout and mutant cell lines

A 4T1 E-cadherin knockout cell line was established using lentiviral-mediated CRISPR/Cas9 methods. Guide RNAs (gRNAs) targeting *Cdh1* were developed by the Lentivirus Construct Core at Brown University and packaged into lentivirus. The gRNA sequence used was GCGGCACCGGGCTCCCATGGCGG. Modified cells were selected using puromycin and knockouts were validated using flow cytometry. E-cadherin knock-in cell lines were established using retroviral overexpression vectors. Plat-E cells were cultured in the presence of 1 μg/mL puromycin and 10 μg/mL blasticidin to maintain expression of retroviral packaging components. E-cadherin constructs (I_160_A, P_161_A, and I_163_A) were outsourced to GenScript, sequenced, and inserted into the unique *BglII* and *HpaI* sites of the retroviral vector MSCV-IRES-GFP (pMIG) (RRID: Addgene_20672). pMIG vectors containing wild-type or mutagenized E-cadherin sequences were introduced using the Lipofectamine LTX Reagent with PLUS (Invitrogen; Cat#15338030) following the provided instructions. Retrovirus produced by Plat-E cells was collected from the supernatant, filtered through a 0.45 μm filter, and used to induce E-cadherin expression in target cells. GFP^+^ cells were sorted via fluorescence-activated cell sorting, and expression of E-cadherin was confirmed via flow cytometry.

### In vivo tumor models

Cells were washed and resuspended in Hanks’ Balanced Salt Solution. For in vivo experiments, cells were lifted with TrypLE (Cat # 12604013). For B16-F10 and derived experiments, unless otherwise noted, 2 × 10^5^ cells were administered in a total volume of 200 μL for intravenous experiments and 100 μL for subcutaneous experiments. For 4T1 and derived experiments, 1 × 10^5^ cells were injected in a volume of 200 μL for intravenous experiments and 100 μL for subcutaneous experiments. For RMA-S and derived experiments, 1 × 10^6^ cells were injected in a volume of 100 μL for subcutaneous injections and 5 × 10^6^ cells were injected in a volume of 200 μL for intravenous injections. For lung tumor nodule quantification, lungs were perfused with PBS containing 1% FBS prior to removal. For in vivo NK cell depletion experiments, mice were injected intraperitoneally with anti-NK1.1 (BioXCell; Cat#BE0036, RRID: AB_1107737) or isotype control IgG2a antibodies (BioXCell; Cat#BE0085, RRID: AB_1107771) (200 μg in 200 μL PBS) on days −2 and −1 relative to tumor injection. Depletion efficiency was confirmed via flow cytometry. Tumor volumes from subcutaneous experiments were measured every third day using precision calipers and calculated using the formula V=(L*W2)/2. Mice were monitored daily and were euthanized when tumor volume exceeded 2,000 mm^3^, weight loss exceeded 20%, or animals became otherwise moribund as required by approved Institutional Animal Care and Use Committee protocol #23-08-0002.

### Isolation of murine lymphocytes

Mice were sacrificed by cervical dislocation following treatment with isoflurane. For subcutaneous experiments, tumors were harvested, cleaned, mechanically dissociated in a GentleMacs C tube, and were fully dissociated in collagenase IV for 45 min at 37 °C. Cells were then passed through a 70 μm filter and washed 2 times before being overlayed onto a 2-step discontinuous Percoll gradient (GE Healthcare Bioscience). Lymphocytes were collected from the Percoll gradient and washed in PBS containing 1% FBS prior to analysis. Spleens were dissociated in 50 mM NH_4_Cl for 10 min, filtered through nylon mesh, and washed 3 times with PBS containing 1% FBS prior to analysis. Cell counts were obtained using trypan blue exclusion on a Countess 3 (Thermo Fisher Scientific) or on a hemocytometer.

### Antibodies and flow cytometry

Adherent cell lines were lifted using 1 mg/mL collagenase type IV and washed prior to staining for flow cytometry to preserve KLRG1-tetramer and cadherin staining. KLRG1-tetramer was produced either in house or by the National Institutes of Health Tetramer Facility (RRID: SCR_026557).^16^ KLRG1-tetramer staining included a 20-min, room-temperature incubation in the dark prior to a 20-min incubation on ice in the dark. When tetramer was not used in the stain set, samples were resuspended in PBS containing 1% FBS and stained for 20 min on ice in the dark. Included in all antibody staining cocktails was an anti-CD16/CD32 (clone 2.4G2; BioXCell; Cat#BE0307, RRID: AB_2736987) blocking antibody and brilliant violet staining buffer (BD Biosciences; Cat#563794). Nuclear staining was performed using the FoxP3 Staining Buffer Set (Miltenyi; Cat#130-093-142). Antibodies used for flow cytometry: from Invitrogen, FoxP3-PE-Cy5.5 (Cat# 35-5773-80; RRID: AB_468806); from BioLegend, PD-1-PE (Cat# 135206; RRID: AB_1877231), E-Cadherin-BV421 (Cat# 147319; RRID: AB_2750483), CD45-BV570 (Cat# 103136; RRID: AB_2562612), and TIM-3-BV785 (Cat# 119725; RRID: AB_2716066); from BD, CD3e-BUV395 (Cat# 563565; RRID: AB_2738278), TCRb-BUV496 (Cat# 749915; RRID: AB_2874154), NK1.1-BUV615 (Cat# 751111; RRID: AB_2875140), KLRG1-BV421 (Cat# 562897; RRID: AB_2737875), CD8-BUV805 (Cat# 612898; RRID: AB_2870186), Ly-108-BV650 (Cat# 740628; RRID: AB_2740323), KLRG1-BV711 (Cat# 564014; RRID: AB_2738542), CD44-FITC (Cat# 553133; RRID: AB_2076224), and CD4-BUV563 (Cat# 612923; RRID: AB_2870208). Cells were run on a BD FACSAriaIII, Cytek Aurora Spectral Flow Cytometer, or BD FACSCelesta, and data were analyzed using FlowJo software (TreeStar; version 10.10, RRID: SCR_008520). For in vivo NK cell depletion, anti-mouse NK1.1 (BioXCell; Cat#BE0036, RRID: AB_1107737) or isotype control IgG2a (Bio X Cell; Cat#BE0085, RRID: AB_1107771) was used. For Western blots, biotinylated anti-β-actin polyclonal antibody (Thermo Fisher Scientific; Cat#600-406-886, RRID: AB_10705204) and purified anti-E-cadherin (BD Cat#610182, RRID: AB_397581) were used. For secondary reagents, HRP-donkey anti-mouse IgG (Jackson ImmunoResearch; Cat#715-036-151, RRID: AB_2340774) and HRP-streptavidin (Jackson ImmunoResearch; Cat#016-030-084, RRID: AB_2337238)

### In vitro proliferation assay

Cells were harvested, washed, counted, and stained in 1 mL CellTrace Violet (CTV) as outlined by the manufacturer’s instructions (Thermo Fisher Scientific; Cat#C34557). Cells were then analyzed by flow cytometry at day 0 to determine initial CTV median fluorescence intensity, then plated and allowed to proliferate for 2 or 3 d when CTV median fluorescence intensity was reassessed.

### Western blot

Samples were separated on 4% to 20% gradient SDS-PAGE gels and transferred to a nitrocellulose membrane (Bio-Rad Laboratories). The membrane was blocked in 5% milk for 1 h at room temperature and incubated with the indicated primary antibody overnight at 4 °C. After washing, the membrane was incubated with secondary reagents for 1 h at room temperature and developed using a ChemiDoc XRS+ System (Bio-Rad).

### Statistical analysis

Analyses for determining statistical significance were done using GraphPad Prism 10 (RRID: SCR_002798; GraphPad Software). Experiment sample sizes and thresholds for significance (*P* values) are noted in figure legends. Unpaired, 2-tailed Student *t* tests were used to compare 2 groups, 1-way analysis of variance was used to compare 3 or more groups with 1 experimental variable (Kruskal-Wallis tests with Dunn’s comparisons tests were used for nonparametric tests), 2-way analysis of variance was used to compare 3 or more groups with 2 experimental variables, and log-rank (Mantel-Cox) tests were used for survival studies. Data represent the mean ± SEM. Sample sizes were based on estimations by power analysis with a level of significance of 0.05 and a power of 0.9.

### Study approval

Animal studies were conducted in accordance with a protocol reviewed and approved by Brown University (Institutional Animal Care and Use Committee protocol #23-08-0002).

## Results

### Generation of a cell line library with variable E-cadherin expression

**Figure 1:**
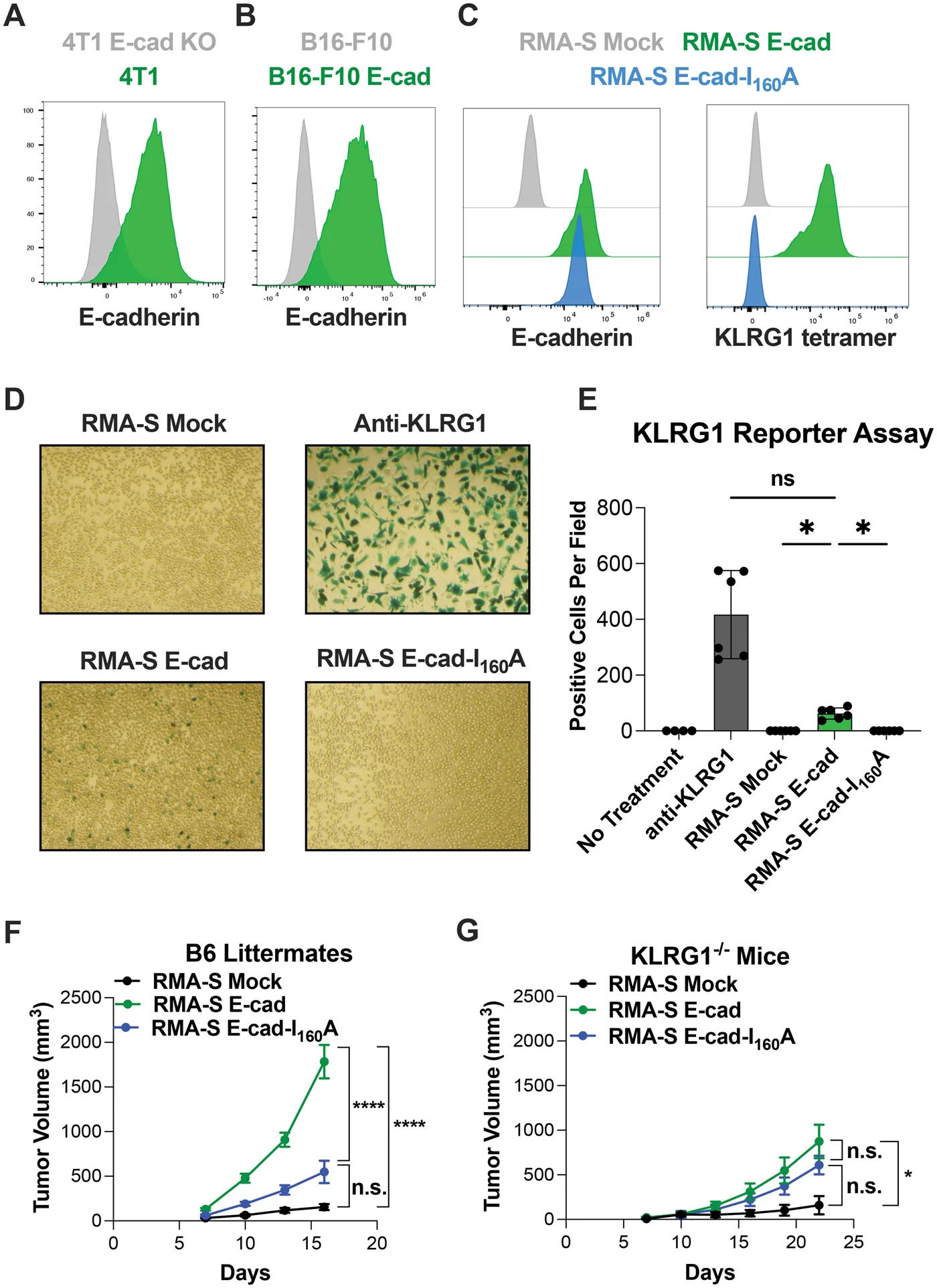
Generation of a cell line library with variable E-cadherin expression. (A) Flow cytometry profile of 4T1 and 4T1 E-cad knockout stained with an anti-E-cadherin monoclonal antibody (mAb). (B) Flow cytometry profile of B16-F10 and B16-F10 E-cad stained with an anti-E-cadherin mAb. (C) Flow cytometry profile of RMA-S, RMA-S E-cad, and RMA-S E-cad-I_160_A stained with an anti-E-cadherin mAb or KLRG1 tetramer. (D, E) KLRG1 reporter cells (BWZ.36.KLRG1) were plated with various activating stimuli (e.g. agonist antibody- or RMA-S–derived cell lines). (D) A representative experiment is shown. (E) Wells were imaged in triplicate and blue cells (i.e. KLRG1-activated cells) were quantified. (F) B6 or (G) KLRG1^−/−^ mice were injected subcutaneously with 1 × 10^6^ RMA-S and derived cell lines. Tumor volumes were measured twice weekly using precision calipers (n = 15–29 for panel F, n = 5–7 for panel G). Data are representative of 2 (A–E and G) or 4 (F) independent experiments. A Kruskal-Wallis test with Dunn’s comparisons test (D) or 1-way analysis of variance with Sidak’s multiple comparisons test (F, G) was performed to determine significance. *P < 0.05, ****P < 0.0001. Error bars represent SEM. n.s., not significant.

To determine how tumor E-cadherin expression influences antitumor responses, we used retroviral overexpression vectors and CRISPR/Cas9 lentiviral systems to develop a library of cell lines with varying E-cadherin expression. The 4T1 mammary carcinoma cell line naturally expresses E-cadherin at high levels; therefore, we deleted E-cadherin from the genome using CRISPR/Cas9 (Fig. 1A). We observed modest gross morphological differences between 4T1 and 4T1 E-cad knockout cells (Fig. S1A); however; we found no difference in proliferation rates between these two cell lines (Fig. S1B, C). The B16-F10 melanoma cell line expresses low levels of N-cadherin but lacks E-cadherin.^26^ B16-F10 E-cad cells express physiological levels of E-cadherin (Fig. 1B), which do not alter the in vitro proliferation rate of B16-F10 melanoma.^26^

To elucidate the role of E-cadherin as a mediator of KLRG1 inhibitory signaling while leaving its function as an adhesion molecule unchanged, we took advantage of the structure of KLRG1 in complex with E-cadherin.^28^ Several amino acids were previously identified as potential critical residues for facilitating interactions with KLRG1 yet were predicted not to affect cadherin-cadherin interactions, which are important for maintaining cell morphology.^28^^,^^29^ We performed an alanine scan of 3 conserved E-cadherin residues between human and mouse that interact with KLRG1 in the complex structure. These included I_160_A, P_161_A, and I_163_A (Fig. S1D). We therefore devised retroviral overexpression vectors and expressed either wildtype E-cadherin (E-cad) or mutated E-cadherins in the RMA-S lymphoma cell line. Using anti-E-cadherin antibodies, we found that E-cadherin expression was comparable in RMA-S E-cad and the 3 mutants (not shown). Although KLRG1 tetramer binding was decreased in all mutant cases (not shown), a total loss of tetramer binding was observed for RMA-S E-cad-I_160_A cells, demonstrating the mutant’s inability to bind KLRG1 (Fig. 1C). Notably, Western blot analysis confirmed E-cadherin expression across all cell lines (Fig. S1E). We did not observe any differences in in vitro proliferation rates between these RMA-S cell lines (Fig. S1F, G). We then validated the functional attributes of these modifications using a BWZ.36.KLRG1 reporter cell line.^16^ In this reporter cell line, a chimeric receptor was engineered by fusing the Ly49H cytoplasmic and transmembrane domains with the KLRG1 extracellular domain, enabling direct signaling upon KLRG1 ligand engagement. Consistent with KLRG1 tetramer staining, we found that KLRG1 signaling is activated by the RMA-S E-cad cells, but not parental RMA-S or RMA-S E-cad-I_160_A cells, further confirming that the E-cadherin mutant cell line cannot engage KLRG1 (Fig. 1D, E).

Last, we further validated the effect of these modifications to RMA-S cells using an in vivo tumor model. Here, we observed E-cadherin–expressing tumors grew significantly more rapidly than their E-cadherin–deficient counterpart (i.e. RMA-S Mock) (Fig. 1F). Though the RMA-S E-cad-I_160_A tumors exhibited slightly faster growth than mock transduced RMA-S, this difference was not statistically significant. Importantly, the RMA-S E-cad-I_160_A tumors exhibited significantly slower growth compared with RMA-S E-cad tumors (Fig. 1F), further supporting the role of KLRG1-E-cadherin interactions in suppressing antitumor immunity. In contrast to B6 mice, in syngeneic KLRG1^−/−^ animals, the growth advantage that RMA-S E-cad has over RMA-S E-cad-I_160_A cells in vivo is not apparent, with both tumors growing at a comparable delayed rate relative to wildtype mice (Fig. 1G). Notably, depletion of NK cells in B6 mice rendered the sensitive RMA-S E-cad-I_160_A line markedly more aggressive, whereas the already highly aggressive RMA-S E-cad line showed a trend toward even greater aggressiveness (Fig. S1H, I). Together, these findings support the conclusion that the protumor effects of E-cadherin are primarily mediated through KLRG1-mediated immune suppression.

### E-cadherin expression enhances tumor aggressiveness

**Figure 2:**
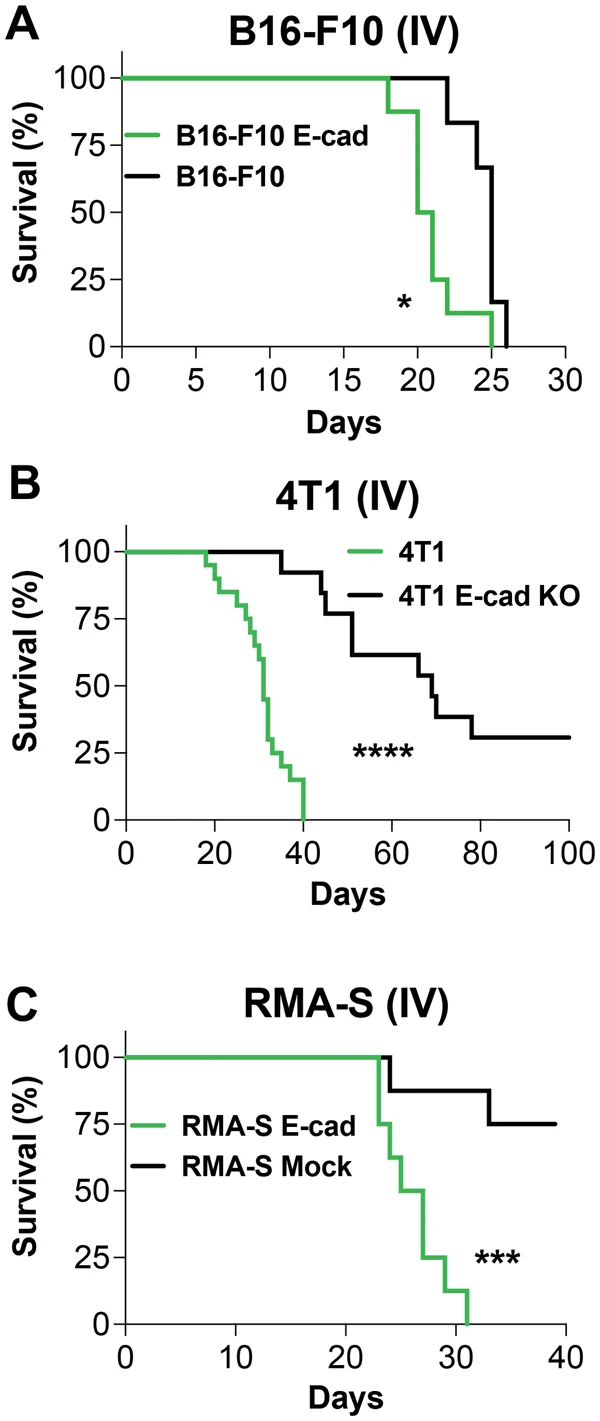
E-cadherin expression renders tumors more aggressive. (A) B6 mice were injected intravenously with 2 × 10^5^ B16-F10 or B16-F10 E-cad. (B) Balb/c mice were injected intravenously with 1 × 10^5^ 4T1 or 4T1 E-cad knockout (KO). (C) B6 mice were injected intravenously with 5 × 10^6^ RMA-S Mock, RMA-S E-cad, or RMA-S E-cadI_160_A mutant. n = 8–20, representing 2 (A, B) or 1 (C) independent experiments. Kaplan-Meier plots depict overall survival, and log-rank (Mantel-Cox) tests were used to determine significance. *P < 0.05, ***P < 0.001, ****P < 0.0001. IV, intravenous.

Although EMT and MET alter transformed cellular behavior and responses to treatment, how varying cadherin expression during EMT/MET impacts cell-extrinsic factors, such as in vivo antitumor responses, remain less well understood.^30–33^ To this end, we sought to determine the in vivo effect of tumor E-cadherin expression on cancer aggressiveness and tumor growth. Following intravenous injection of each cell line tested, E-cadherin expression consistently promoted more aggressive disease, as seen by significantly reduced survival rates (Fig. 2A–C). This is particularly evident in the highly invasive 4T1 model, which remains fully controlled until day 40 in the absence of E-cadherin expression, with 25% of the mice surviving at day 100 post–intravenous injection (Fig. 2B). Taken together, these results indicate that E-cadherin expression confers a robust tumor growth advantage across models, with protumor effects largely attributable to KLRG1 engagement.

### KLRG1 and PD-1 are variably coexpressed on tumor-infiltrating lymphocytes in E-cadherin^+^ B16-F10 melanoma

**Figure 3:**
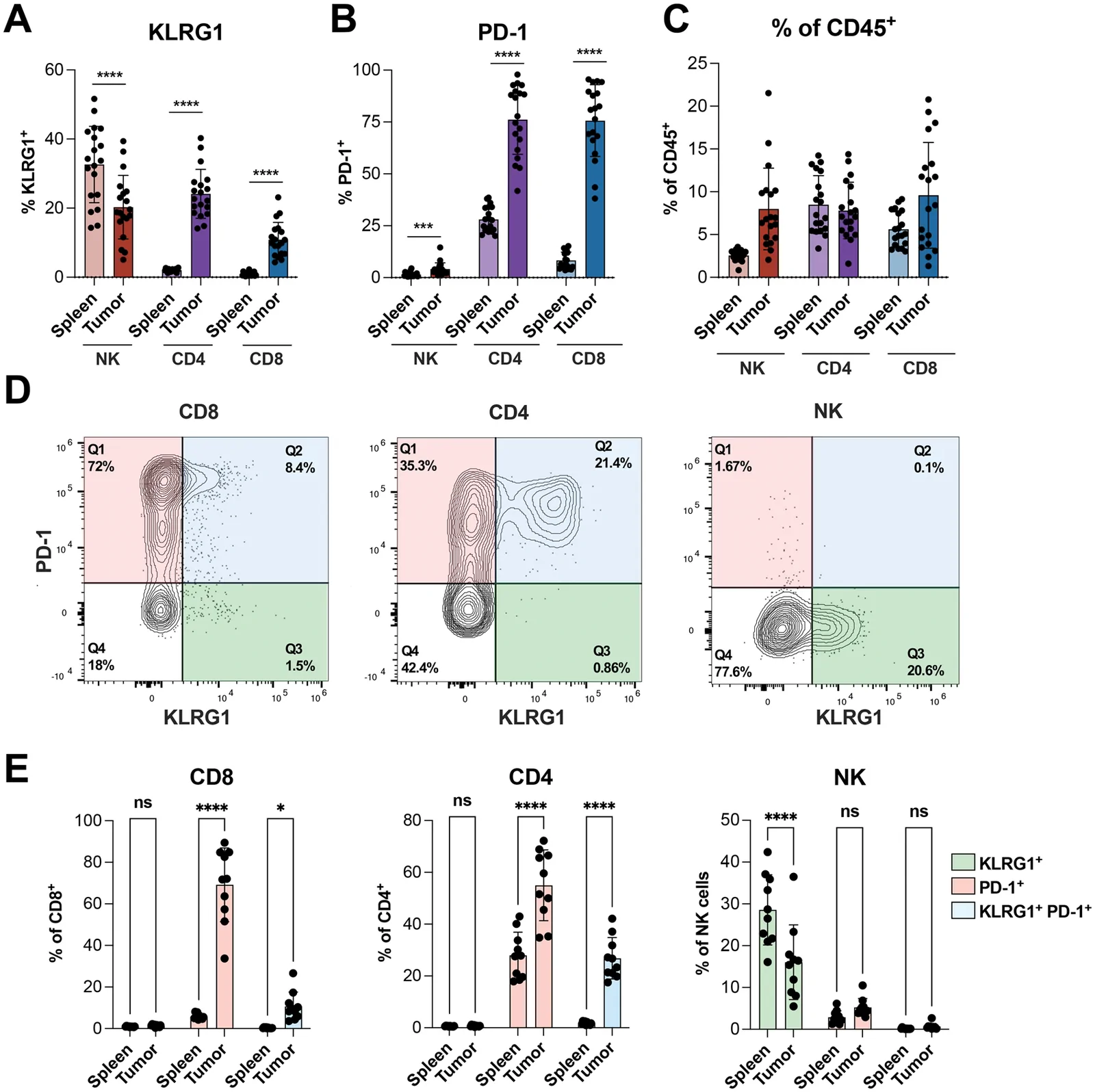
KLRG1 and PD-1 are variably coexpressed on tumor-infiltrating lymphocytes in E-cadherin^+^ B16-F10 melanoma. (A–G) B6 mice were injected subcutaneously with 2 × 10^5^ B16-F10 E-cad, and tissues were harvested 16 d later. (A) KLRG1 and (B) PD-1 expression was quantified on splenic and tumor-infiltrating lymphocytes (n = 19, pooled from 3 independent experiments). (C) Population subsets as percentages of total CD45^+^ cells were quantified. (D, E) Coexpression of KLRG1 and PD-1 was assessed on tumor-infiltrating CD8^+^ T cells, CD4^+^ T cells, and NK cells. Two-way analysis of variance with Sidak’s multiple comparisons tests were performed to determine significance. *P < 0.05, ***P < 0.001, ****P < 0.0001. Error bars represent SEM. ns, not significant.

Following tumor infiltration, effector lymphocytes upregulate checkpoint inhibitory receptors in various combinations that often correlate with dysfunction. To examine KLRG1 expression patterns on tumor-infiltrating lymphocytes in our model, we injected mice subcutaneously with B16-F10 or B16-F10 E-cad and harvested tumors after 16 d of growth. As described previously,^26^ B16-F10 E-cad tumors are infiltrated by both T and NK cells (Fig. 3; Fig. S2 for gating strategy). We did not observe any quantitative differences in immune cell infiltration between B16-F10 and B16-F10 E-cad tumors, nor did we observe differences in lymphocyte KLRG1 or PD-1 expression between tumors (Fig. S3A–C). However, CD8^+^ T cells from B16-F10 E-cad tumors were composed of significantly more terminally exhausted cells (CD44^+^ PD-1^+^ Ly-108^−^ TIM-3^+^) and significantly fewer progenitor exhausted cells (CD44^+^ PD-1^+^ Ly-108^+^ TIM-3^−^) relative to B16-F10 tumors (Fig. S3D, E). In B16-F10 E-cad tumors, relative to splenic populations in the same mice, we observed that both tumor-derived CD4^+^ and CD8^+^ T cells upregulate KLRG1 expression to varying degrees (Fig. 3A). Approximately 11% of tumor-infiltrating CD8^+^ T cells expressed KLRG1, compared with ∼1% of splenic CD8^+^ T cells (Fig. 3A). Notably, to our knowledge, this level of KLRG1 expression on CD8^+^ T cells has not been reported in a noninfection model. This finding may explain the predominant role of KLRG1 on T cells rather than NK cells, which we observed in the B16-F10 E-cad experimental metastasis model shown subsequently. We did not observe appreciable PD-1 expression on NK cells, and these few PD-1^+^ cells rarely coexpressed KLRG1 (Fig. 3B). Unlike NK cells, the large majority of infiltrating T cells expressed PD-1 at this time point (Fig. 3B). Notably, within the tumors, NK cells, CD4^+^ cells, and CD8^+^ T cells, each comprise a comparable proportion of CD45^+^ cells (Fig. 3C). Importantly, the majority of KLRG1^+^ T cells also expressed PD-1 (Fig. 3D, E), supporting the synergy reported by us and others during dual blockade.^26^^,^^34^ Taken together, these data reveal distinct KLRG1 expression patterns on tumor-infiltrating lymphocyte subsets, with unexpectedly high expression on CD8^+^ T cells and coexpression with PD-1, supporting a role for KLRG1 in modulating adaptive anti-tumor responses that may have previously been underestimated.

### Tumor E-cadherin expression modulates sensitivity to innate and adaptive immune control

**Figure 4:**
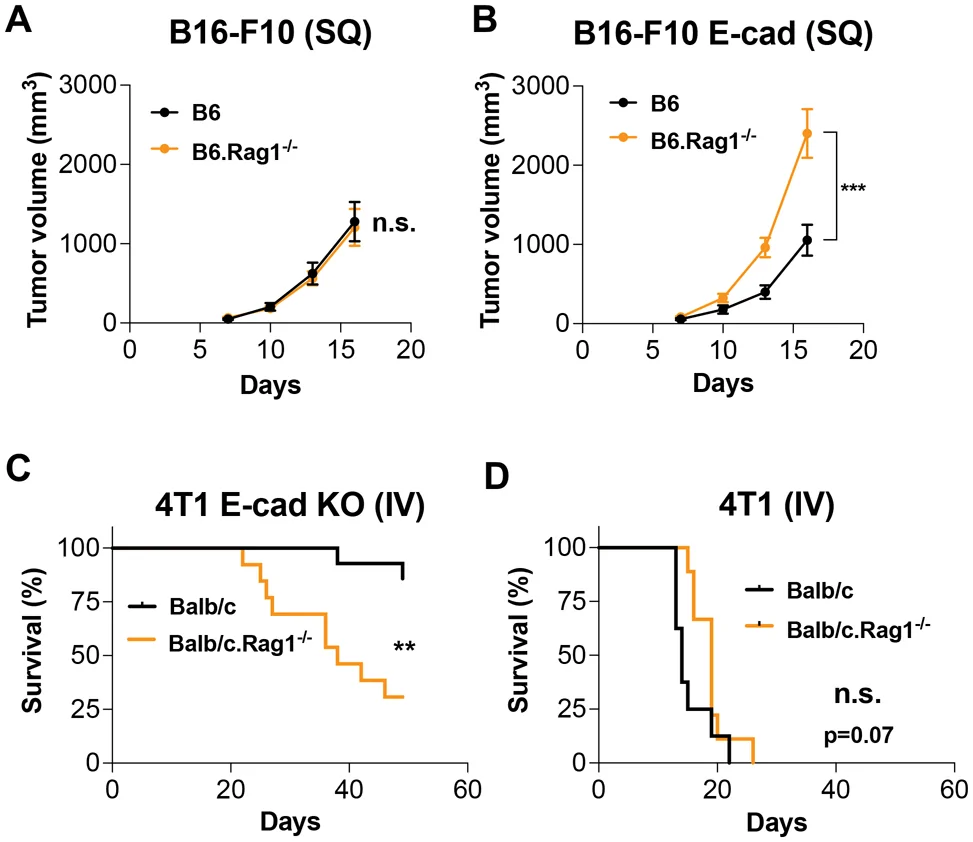
Tumor E-cadherin expression modulates sensitivity to innate and adaptive immune control. (A, B) B6 and syngeneic B6.Rag1^−/−^ mice were injected subcutaneously with 2 × 10^5^ B16-F10 or B16-F10 E-cad. Tumor volumes were measured twice weekly using precision calipers (n = 9–11, pooled from 2 (A) or 3 (B) independent experiments). (C, D) Balb/c and syngeneic Balb/c-Rag1^−/−^ mice were injected intravenously with 1 × 10^5^ 4T1 E-cad knockout (C) or 4T1 (D) and assessed for survival (n = 12–16, pooled from 2 independent experiments). Kaplan-Meier plots depict overall survival, log-rank (Mantel-Cox) tests were performed to determine significance. **P < 0.01, ***P < 0.001. IV, intravenous; n.s., not significant; SQ, subcutaneous.

Given the varied immune infiltrates of B16-F10 E-cad tumors and the robust protumor effects of tumor E-cadherin expression, we sought to evaluate how E-cadherin expression alters tumor sensitivity to innate and adaptive immune control. For our melanoma model, we injected B16-F10 and B16-F10 E-cad tumors subcutaneously in B6 and syngeneic *Rag1*^−/−^ mice. For unmodified B16-F10, the absence of an adaptive immune response had no impact on tumor growth rate as both B6 and *Rag1*^−/−^ mice exhibited similar disease progression rates (Fig. 4A). This is in line with previous studies showing B16-F10 melanoma’s low T cell immunogenicity but pronounced sensitivity to NK cells.^35^^,^^36^ However, for the B16-F10 E-cad group, *Rag1*^−/−^ mice exhibited significantly more rapid tumor growth, revealing that an intact adaptive immune system is required for control of B16-F10 melanoma when E-cadherin is expressed (Fig. 4B). To extend this investigation into an additional strain of mice and tumor model, we used the 4T1 and 4T1 E-cad knockout tumor models in Balb/c and syngeneic *Rag1*^−/−^ animals. When 4T1 E-cad knockout tumors were injected intravenously, the Rag1-deficient cohort exhibited significantly shorter survival compared with their immunocompetent counterparts (Fig. 4C). This suggests that in the absence of E-cadherin, the adaptive immune system is crucial for controlling tumor progression. In contrast, following injection of parental 4T1, which robustly expresses E-cadherin, both groups rapidly succumbed to disease at similar rates (Fig. 4D). These findings suggest that E-cadherin expression can, in some cases, drive a moderately aggressive tumor to bypass the defenses of an intact immune system. Collectively, these data reveal the tumor-specific alterations that E-cadherin expression can confer on transformed cells and underscore the distinct contributions of innate and adaptive immune responses in these models.

### Klrg1 deletion in CD8^+^ T cells enhances control of E-cadherin^+^ tumors

**Figure 5:**
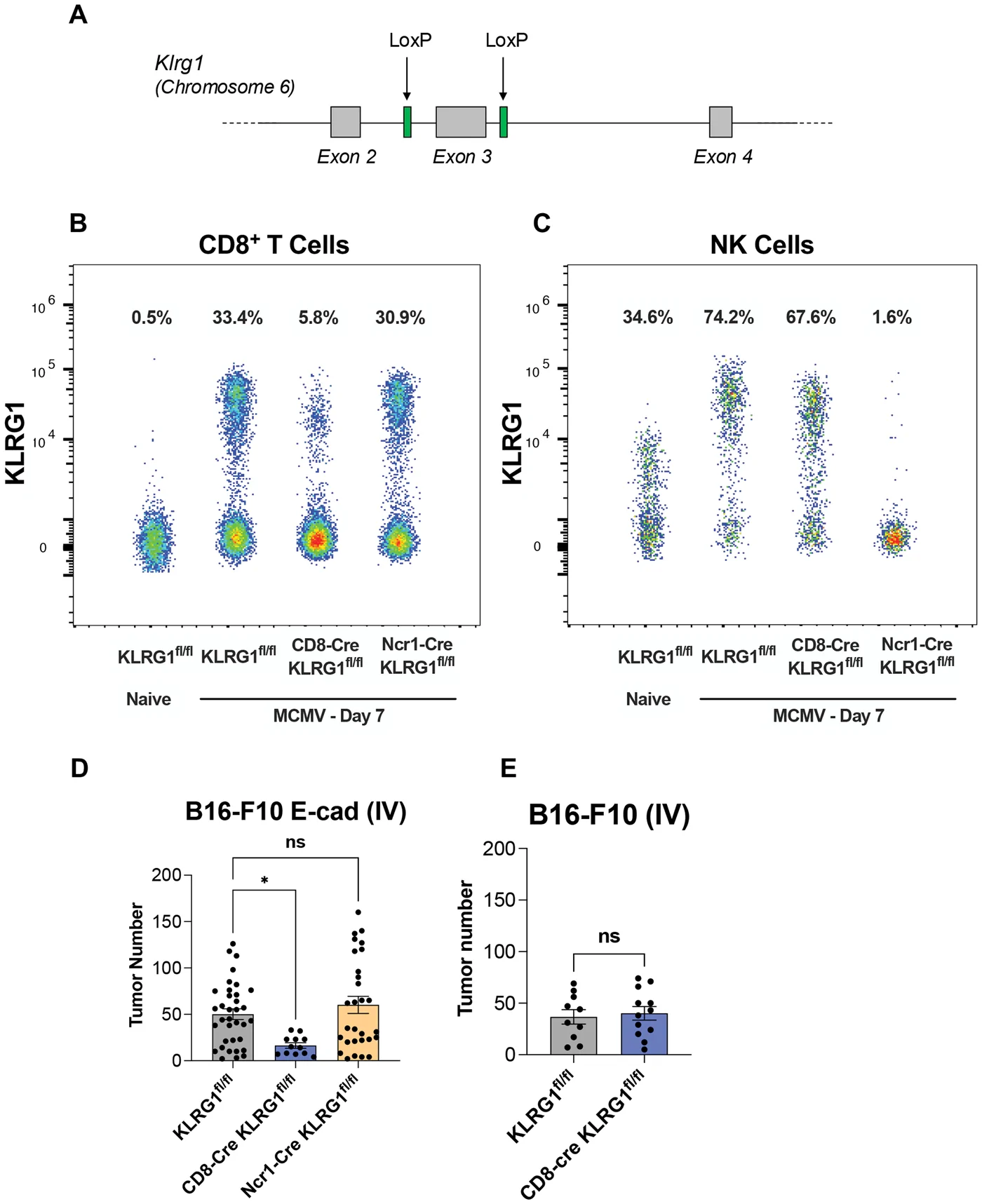
*Klrg1* deletion in CD8^+^ T cells enhances control of E-cadherin^+^ tumors. (A) KLRG1^fl/fl^ mice were generated using CRISPR/Cas9 to target exon 3 with LoxP sites on the 5′ (155 bp upstream) and 3′ (93 bp downstream) flanks. (B, C) Mice were infected with MCMV, and spleens were harvested on day 7 postinfection to evaluate KLRG1 expression on CD8^+^ T cells (B) and NK cells (C) via flow cytometry. (D) CD8-Cre-KLRG1^fl/fl^ (n = 14), Ncr1-Cre-KLRG1^fl/fl^ (n = 18), and KLRG1^fl/fl^ littermate control animals (n = 41) were injected intravenously with 2 × 10^5^ B16-F10 E-cad. On day 21, mice were sacrificed, lungs were perfused, and tumors were quantified macroscopically. (E) CD8-Cre-KLRG1^fl/fl^ (n = 12) and KLRG1^fl/fl^ (n = 10) littermate control animals were injected intravenously with 2 × 10^5^ B16-F10. On day 21, mice were sacrificed, lungs were perfused, and tumors were quantified macroscopically. Data are pooled from 8 (D) or 2 (E) independent experiments. One-way analysis of variance with Sidak’s multiple comparisons test (D) or Student’s paired *t* test was performed to determine significance. *P < 0.05. Error bars represent SEM. n.s., not significant.

Our laboratory previously demonstrated that KLRG1^−/−^ mice exhibit a higher degree of tumor control in a model of experimental metastasis using B16-F10 E-cad tumor cells.^26^ However, due to a lack of in vivo genetic tools, we could not identify which KLRG1-expressing populations of immune cells were mediating the observed immune suppression. To address this issue, we generated KLRG1^fl/fl^ animals using CRISPR/Cas9-mediated homology-directed repair, inserting loxP sites on each side of the targeted exon (exon 3) (Fig. 5A). KLRG1^fl/fl^ animals were then crossed to Ncr1-Cre or E8i-Cre (referred to as CD8-Cre) to delete *Klrg1* in NK cells and CD8^+^ T cells, respectively.^37^^,^^38^ We validated the specificity of recombination using MCMV infection, and for each new strain generated, the recombination appeared specific to each immune population (Fig. 5B, C). Notably, while Ncr1-Cre results in nearly complete *Klrg1* deletion in NK cells (>98%) (Fig. 5C), a small percentage (∼6%) of CD8^+^ T cells escape deletion when CD8-Cre is used (Fig. 5B). Nevertheless, by crossing KLRG1^fl/fl^ mice to the well-described CD8-Cre and Ncr1-Cre strains, we generated animals with tissue-specific deletions of *Klrg1* in vivo. Importantly, KLRG1 is not deleted from conventional CD4^+^ T cells or FoxP3^+^ Treg cells in the CD8-Cre KLRG1^fl/fl^ mice (data not shown). To test the role of KLRG1 signaling in antitumor immunity, we injected B16-F10 E-cad cells intravenously into CD8-Cre KLRG1^fl/fl^, Ncr1-Cre KLRG1^fl/fl^, and KLRG1^fl/fl^ littermate control animals, and harvested lungs after 21 d to enumerate tumor nodules. In the CD8-Cre KLRG1^fl/fl^ mice, but not in the Ncr1-Cre KLRG1^fl/fl^ group, we observed significantly fewer lung metastases than the control (Fig. 5D). We did not observe the same trend when parental B16 (E-cadherin–negative) was engrafted, again underscoring the KLRG1-dependent protumor effect of E-cadherin (Fig. 5E). Thus, these data demonstrate that KLRG1 expression on CD8^+^ T cells, but not NK cells, promotes increased metastasis of E-cadherin^+^ tumors to the lung. Taken together, our results across all 3 models show that E-cadherin expression increases tumor aggressiveness by weakening CD8^+^ T cell responses (Figs. 4B, C and 5D) as well as NK cell responses (Fig. 1F).

## Discussion

The loss of E-cadherin expression on transformed cells is a hallmark of the EMT and is commonly linked with tumor metastasis.^3^ However, recent findings on the prometastatic effects of E-cadherin and its role in the MET challenge the universality of this paradigm. First, clinical studies have shown that invasive carcinomas frequently express E-cadherin in both primary tumors and metastases, with multiple reports indicating that its expression is associated with poor overall survival.^7–12^ Additionally, preclinical in vivo studies have identified several tumor-promoting roles for E-cadherin that illuminate the molecular basis for these effects.^14^^,^^39–41^ For example, using Cre-lox in vivo conditional deletions of *Cdh1*, the gene encoding E-cadherin, Padmanaban et al.^14^ uncovered a nuanced mechanism whereby E-cadherin weakens tumor invasiveness but enhances cancer cell proliferation, survival, and metastatic outgrowth. Specifically, E-cadherin–mediated tumor growth was found to rely on TGF-β signaling and reactive oxygen species in the tumor microenvironment.^14^ More recently, in vivo studies have identified changes to cancer cell metabolism and direct interactions with epidermal growth factor receptor as additional mechanisms by which E-cadherin promotes the spread of metastatic cancer, highlighting the multifaceted effects of tumor E-cadherin expression.^39^^,^^40^ Nevertheless, existing studies have yet to fully investigate the direct immunomodulatory functions of tumor-derived E-cadherin, particularly in the context of inhibitory KLRG1 signaling.

In this work, we advance this understanding using a cell line library modified for variable E-cadherin expression and KLRG1 binding affinity. In accordance with its described protumor role, expression of E-cadherin significantly increased in vivo cancer aggressiveness across all cell lines tested (Fig. 2A–C). The effects were most striking for the 4T1 and RMA-S models, likely owing to their robust degree of E-cadherin expression (Figs. 1F and 2B, C). The comparatively modest change in survival rate for B16-F10 E-cad may be due to the fact that parental B16-F10 cells endogenously express N-cadherin, whereas RMA-S and 4T1 cells do not, thus precluding a binary comparison between cadherin-negative and cadherin-expressing states for the B16-F10 model (Fig. 2A). Our finding that RMA-S E-cad-I_160_A tumors grew significantly more slowly than their RMA-S E-cad counterparts, despite identical cadherin expression, underscores a robust immune inhibitory effect for KLRG1 (Fig. 1F, G), and allows us to uncouple KLRG1 binding from cadherin-cadherin homotypic interactions. Notably, isoleucine at position 160 is present in both mouse E-cadherin and N-cadherin and is conserved in humans.^28^ Previously, our laboratory and others have demonstrated that single-agent KLRG1 blockade can enhance control of 4T1 metastasis to the lung and that combination blockade targeting KLRG1 and PD-1 can significantly slow the growth of 4T1, B16-F10, and B16-F10 E-cad tumors in vivo.^26^^,^^34^ This conclusion builds upon and expands previous findings from our laboratory and others, demonstrating that inhibiting KLRG1 signaling can improve immune control of E-cadherin-expressing tumors.^26^^,^^34^

From a clinical perspective, targeting KLRG1 using checkpoint blockade has become increasingly attractive. KLRG1 has historically been considered a feature of effector and senescent CD8^+^ T cells, rather than an exhaustion marker like PD-1 or TIM-3.^42^ However, recent studies challenge this notion and suggest that KLRG1 signaling may dampen CD8^+^ T cell antitumor immunity in a manner similar to PD-1. For example, in a cohort of non-small cell lung cancer patients with E-cadherin^+^ tumors, KLRG1 was found to mark highly cytotoxic CD8^+^ T cells in the blood, yet its expression in the tumor marked exhausted, rather than effector, T cell populations. Moreover, this study found that E-cadherin expression negatively correlated with prognosis in patients with high KLRG1 expression, suggesting that KLRG1 may be contributing to immunosuppression.^43^ Interestingly, for tumor-infiltrating CD8^+^ and CD4^+^ T cells, nearly all of the KLRG1^+^ cells coexpress PD-1 (Fig. 3D, E). This is somewhat surprising, given that expression of these receptors is often mutually exclusive and that the transcription factors that control KLRG1 and PD-1 expression, T-bet and TOX, respectively, generally operate in distinct cell states.^44^^,^^45^ Nevertheless, coexpression of PD-1 and KLRG1 on T cells has been observed in some cancer patients and can explain the synergistic effect of dual therapy that we and others observed.^26^^,^^34^^,^^43^

In summary, our findings reveal a strong protumor effect of E-cadherin expression across a variety of murine cancers and demonstrate that this effect is largely driven by KLRG1 engagement. Using immunodeficient mice, we highlight the variable contributions of innate and adaptive immune populations, which depend on the tumor type. Finally, using tissue-specific conditional knockout mice in a model of E-cadherin–expressing melanoma, we identify a potent antitumor effect of KLRG1 deficiency in CD8^+^ T cells, establishing a novel approach to enhance the immune response against E-cadherin^+^ tumors.

## Supplementary Material

vkag046_Supplementary_Data

## Data Availability

No large datasets or coding scripts were generated from this work. Original data are available upon reasonable request from the corresponding author.
