# Supplementary material for: E-cadherin expression promotes tumor growth via KLRG1-dependent pathways
Source: J Immunol. 2026 Apr 24;215(4):vkag046. doi: 10.1093/jimmun/vkag046 (PMC13108842; doi:10.1093/jimmun/vkag046)
Supplement: vkag046_Supplementary_Data [file vkag046_supplementary_data.pdf]

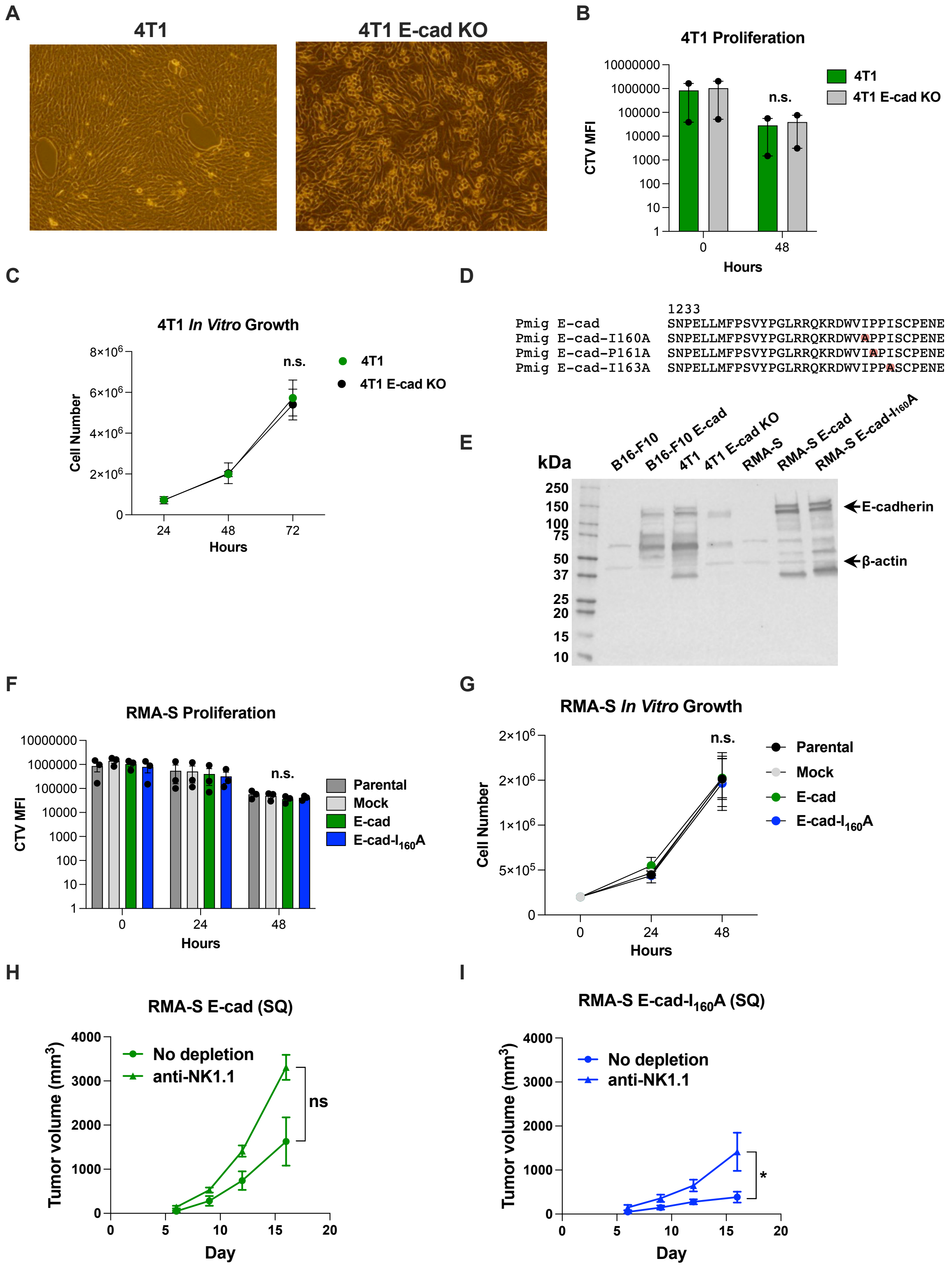

Supplementary Figure 1. Acquisition or loss of E-cadherin does not affect the proliferation potential of tumor cell lines. (A) 4T1 and 4T1 E-cad KO cells were cultured and imaged at 10x magnification using an Olympus DP70. (B-C) 4T1 and 4T1 E-cad KO cells were stained with CellTrace Violet (CTV) and cultured for 72 hours to evaluate proliferation (B) via flow cytometry and cell growth (C) using trypan blue exclusion on a Countess 3. (D) Amino acid sequence alignment for Pmig E-cadherin vector sequences was performed using Benchling. (E) SDS-PAGE immunoblot of E-cadherin was performed using cell lysates from RMA-S, 4T1, and B16 derived tumor cell lines. Beta-actin was used as an internal loading control, representative of three experiments. (F-G) RMA-S and derived cells were stained with CellTrace Violet (CTV) and cultured for 48 hours to evaluate proliferation (F) via flow cytometry and cell growth (G) using trypan blue exclusion on a Countess 3. (H-I) On days -2 and -1 relative to tumor injection, mice were injected intraperitoneally with an isotype antibody or an anti-NK1.1 antibody. Mice were then injected subcutaneously with $1x10^6$ RMA-S E-cad (H) or RMA-S E-cad-I_{160}A cells (I). Data represent two (A-C), three (E-G), and one (H-I, n=7), independent experiment(s). Student's paired t-tests (H-I) or two-way ANOVA with Tukey's multiple comparisons test (B-C, F-G) was performed to determine significance; *p<0.05, ns, not significant. Error bars represent S.E.M.

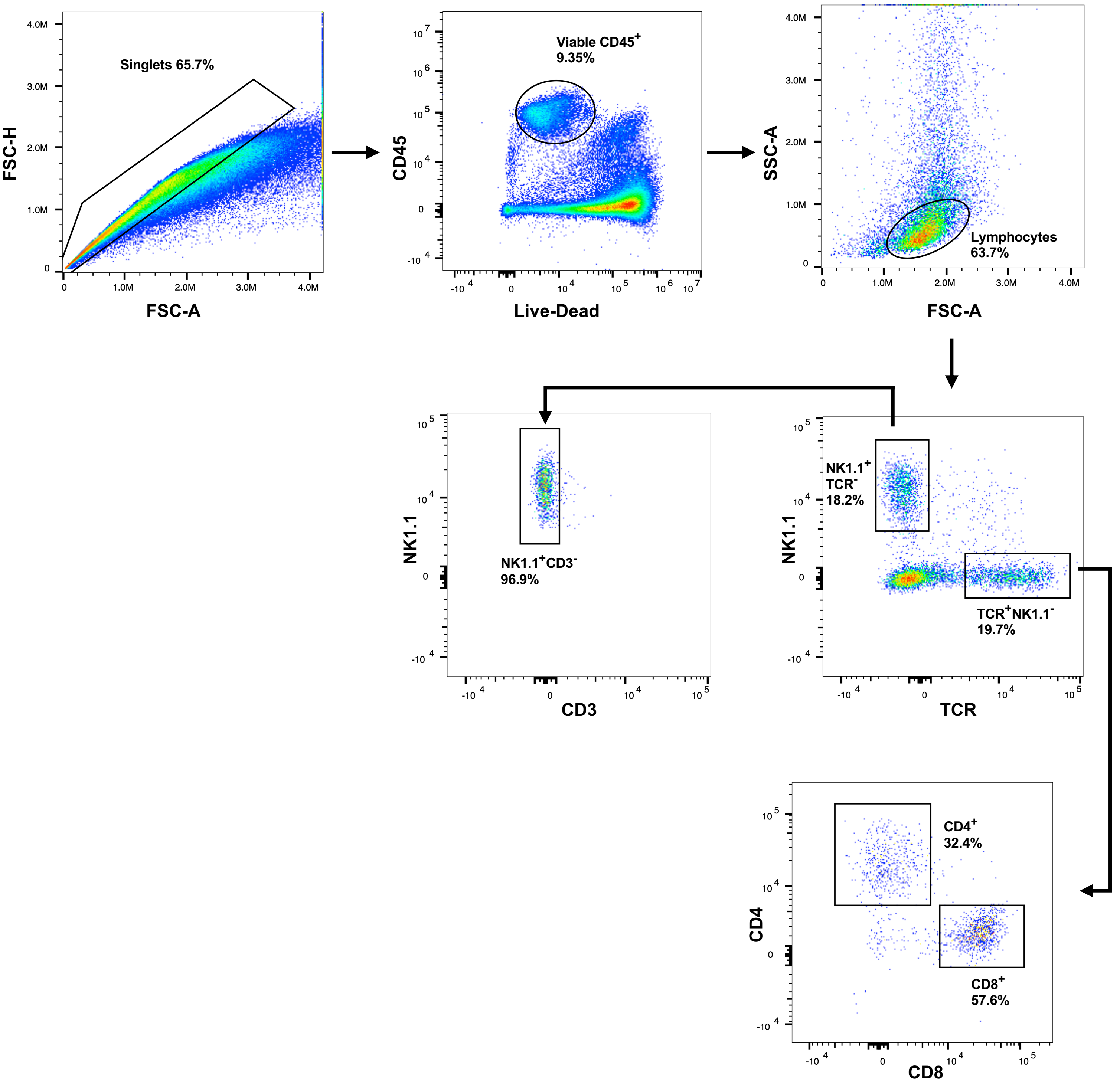

Supplementary Figure 2. Gating strategy for various populations of tumor-infiltrating lymphocytes. Lymphocytes were gated on singlets (FSC-A, FSC-H), viable CD45$^+$ (Live/Dead Zombie NIR, CD45$^+$), and lymphocytes (FSC-A, SSC-A). Then B cells (CD19$^+$), NK cells (NK1.1$^+$, TCR$^-$, CD3$^-$), CD8$^+$ T cells (NK1.1$^-$, TCR$^+$, CD8$^+$), and CD4$^+$ T cells (NK1.1$^-$, TCR$^+$, CD4$^+$) were gated.

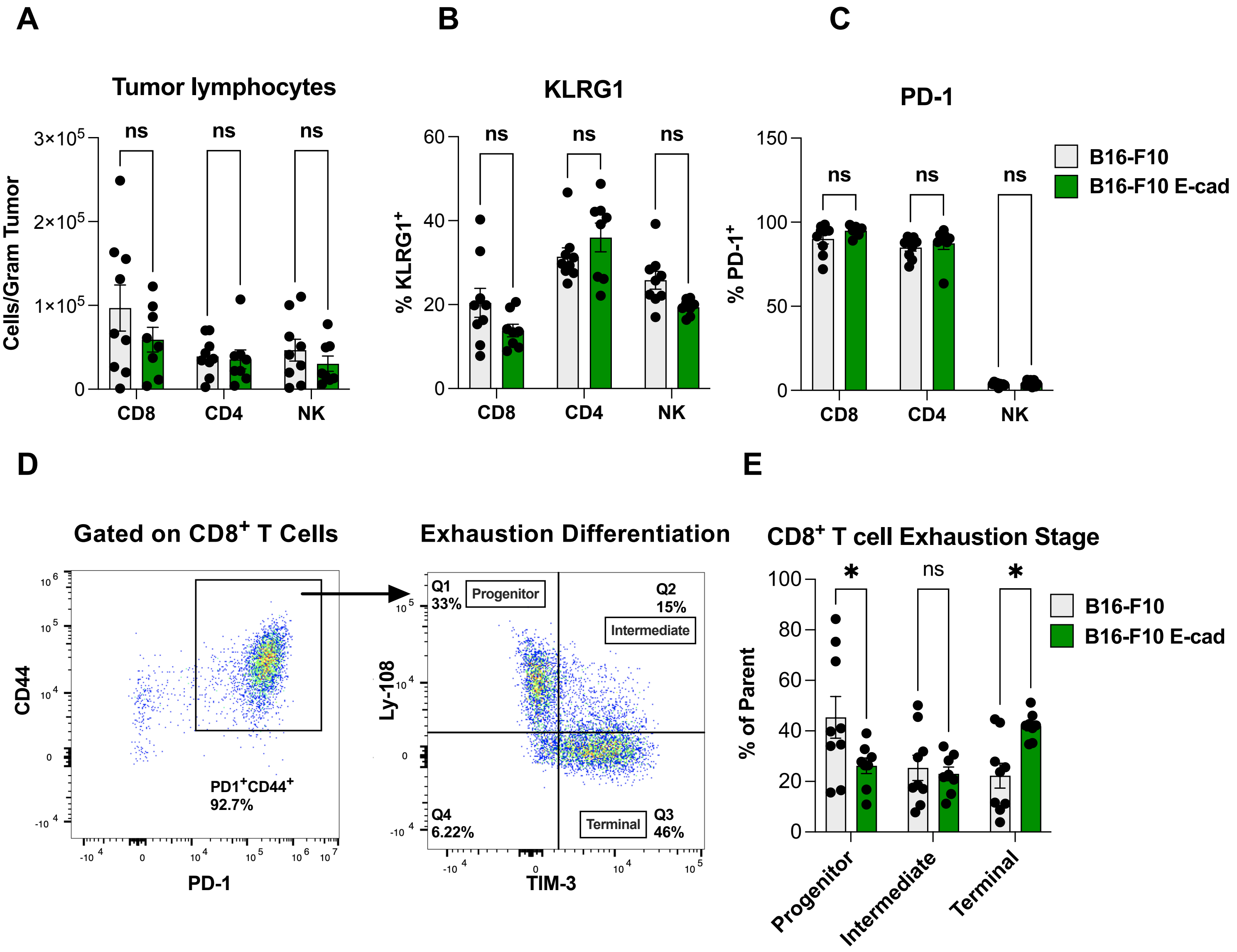

Supplementary Figure 3. CD8$^+$ T cells from B16-F10 E-cad tumors are composed of significantly more terminally exhausted cells. (A-E) B6 mice were injected s.c. with 2x10^5 B16-F10 or B16-F10 E-cad and tumors were harvested 16 days later. Lymphocytes per gram of tumor (A), KLRG1 expression (B), and PD-1 expression (C) were quantified for tumor-infiltrating lymphocytes (n=8-9). (D-E) CD8$^+$ T cells were evaluated for stage of exhaustion. CD8$^+$ T cells were pre-gated for CD44$^+$PD-1$^+$ cells. Next, progenitor (Ly-108$^+$TIM-3$^-$), intermediate (Ly-108$^+$TIM-3$^+$), and terminally exhausted (Ly-108$^-$TIM-3$^+$) cells were analyzed, as the frequency of total CD44$^+$PD-1$^+$CD8$^+$ T cells. Representative pseudo-color plots (D) and cumulative data (E) were generated. Two-way ANOVA with Sidak's multiple comparisons tests were performed to determine significance (A-C, E); *p<0.05, n.s. not significant. Error bars represent S.E.M.
